# The Application of Optical Coherence Tomography Angiography in Cerebral Small Vessel Disease, Ischemic Stroke, and Dementia: A Systematic Review

**DOI:** 10.3389/fneur.2020.01009

**Published:** 2020-09-10

**Authors:** Jun-Fang Zhang, Stewart Wiseman, Maria C. Valdés-Hernández, Fergus N. Doubal, Baljean Dhillon, Yun-Cheng Wu, Joanna M. Wardlaw

**Affiliations:** ^1^Department of Neurology, Shanghai General Hospital, Shanghai Jiao Tong University School of Medicine, Shanghai, China; ^2^Centre for Clinical Brain Science, Edinburgh Imaging and UK Dementia Research Institute, University of Edinburgh, Edinburgh, United Kingdom; ^3^Princess Alexandra Eye Pavilion, NHS Lothian, Edinburgh, United Kingdom

**Keywords:** dementia, Alzheimer's disease, retinal vasculature, optical coherence tomography angiography, ischemic stroke, cerebral small vessel disease

## Abstract

**Objective:** To investigate the application of optical coherence tomography angiography (OCTA) in cerebral small vessel disease (SVD), ischemic stroke and dementia.

**Methods:** We conducted a systematic search in MEDLINE (from inception) and EMBASE (from 1980) to end 2019 for human studies that measured retinal parameters in cerebral SVD, ischemic stroke, and dementia using OCTA.

**Results:** Fourteen articles (*n* = 989) provided relevant data. Ten studies included patients with Alzheimer disease (AD) and mild cognitive impairment (*n* = 679), two investigated pre-symptomatic AD participants (*n* = 154), and two investigated monogenic SVD patients with cerebral autosomal dominant arteriopathy with subcortical infarcts and leukoencephalopathy (*n* = 32) and Fabry disease (*n* = 124). Methods to reduce bias and risk factor adjustment were poorly reported. Substantial methodological variations between studies precluded a formal meta-analysis. Quantitative measurements revealed significant yet inconclusive changes in foveal avascular zone, perfusion density, and vessel density (VD) in AD, presymptomatic AD, and SVD patients. Two (*n* = 160) of three studies (*n* = 192) showed association between decreased VD and increased white matter hyperintensities. In three (*n* = 297) of seven studies (*n* = 563), better cognitive function was associated with increased VD. One study (*n* = 52) suggested increased VD was associated with increased ganglion cell–inner plexiform layer thickness in AD yet with no covariate adjustment.

**Conclusions:** Changes in retinal microvasculature identified using OCTA are associated with monogenic SVD and different stages of AD, but data are limited and partly confounded by methodological differences. Larger studies with risk factors adjustment and more consistent OCTA methods are needed to fully exploit this technology.

**PROSPERO registration number:** CRD42020166929.

## Introduction

Cerebral small vessel disease (SVD) is an intrinsic disorder that affects the brain's small perforating arterioles, capillaries, and probably venules, causing various lesions seen on pathological examination or brain imaging with magnetic resonance imaging (MRI) or computed tomography (1). Cerebral SVD is a common condition in older adults and causes a wide array of clinical syndromes, including 25% of ischemic strokes and 80% of intracerebral hemorrhages, and contributes to up to 50% of dementias worldwide (1–3).

However, the changes in the cerebral microcirculation are difficult to visualize *in vivo*. Because the retinal microvasculature and cerebral microvasculature share similar embryologic origins as well as anatomical and physiological properties (4), investigating the network of retinal vessels may provide new insights into cerebrovascular disease and the vascular contribution to pathologic features of dementia (5). Previous literature has mainly focused on retinal fundus photography or optical coherence tomography (OCT) (5, 6).

A newly presented extension of structural OCT, referred to as optical coherence tomography angiography (OCTA), represents a novel non-invasive, depth-selective modality that allows for visualization of retinal blood flow without dye injection (7). OCTA images are essentially motion-contrast images, which are based on the different backscattering of light between red blood cells and neurosensory tissue, because red blood cells are moving while neurosensory tissue is static (8). In many cases, these images are now approaching histology-level resolution (8), raising great interest in the possibility of detecting early microvascular pathological changes in people *in vivo*. A comprehensive review of the technology, its principles, limitations, and the clinical application in eye disease has been published (8), work that we extend here into the arena of neurological disease. OCTA may permit detection of a series of changes such as reduction in capillary vessels and perfusion density before they are visible on retinal photographs (9). Moreover, recent work suggests that high-resolution OCTA images combined with machine learning may provide great potential to automate detection and quantification of microvascular changes underlying common brain diseases such as SVD, stroke, and dementia, in the future (10, 11).

In this study, we aimed to conduct a systematic review of the literature to examine the application of OCTA in cerebral SVD, ischemic stroke, and dementia, including Alzheimer disease (AD), vascular dementia, frontotemporal dementia, and dementia with Lewy bodies. Specifically, our primary objective is to find out whether there are any specific changes seen in patients with cerebral SVD, ischemic stroke, and/or dementia using OCTA when compared with healthy individuals. Our secondary objective is to assess the association between OCTA changes and brain imaging findings, cognitive functions, and other retinal parameters using fundus camera imaging or standard OCT.

## Methods

This systematic review was based on a predefined protocol (PROSPERO registration no. CRD42020166929) following the Preferred Reporting Items for Systematic Reviews and Meta-analyses (12) guidelines. We refer to SVD MRI findings according to STRIVE (Standards for Reporting Vascular Changes on Neuroimaging) guidelines (13). As all analyses here are based on publicly available summary statistics and not individual-level data, no ethical approval or informed patient consent was required.

### Search Strategy

We conducted a literature search of the Medical Literature Analysis and Retrieval System Online (MEDLINE, from inception) and the Excerpta Medica Database (EMBASE, from 1980) up to December 31, 2019, using the Ovid Web Gateway (detailed search strategy in Supplementary Material). We considered only studies published in English. References of relevant articles were hand-searched, and a forward citation search was performed to identify further studies.

### Inclusion and Exclusion Criteria

The studies included patients with cerebral SVD, ischemic stroke, and/or any type of dementia and compared either (1) retinal parameters using OCTA vs. control, (2) OCTA parameters and brain imaging findings, (3) OCTA parameters and cognitive function, or (4) OCTA parameters and findings on fundus camera imaging or OCT.

The following studies were excluded: (1) duplicate publications or studies not meeting the inclusion criteria, (2) review studies, (3) single-case reports, (4) nonhuman studies, (5) non–English language studies, (6) conference presentations or summaries, (7) studies without details of diagnosis criteria or definitions, and (8) studies that despite examining retinal parameters did not conduct or report results on OCTA.

### Data Extraction

We initially screened all studies identified in the systematic search by abstract and title for potentially relevant articles. Duplicate articles were removed, and the remaining articles were assessed for eligibility after full-text review. Data extracted from these studies included title; authors; publication year; study aim; study type; number of patients and controls; number of male and female, mean age, participant selection criteria, and diagnostic criteria; type of OCTA device; assessment of OCTA image quality; whether OCTA associations were adjusted for demographics, other risk factors, or other covariates; and outcomes. All data were cross-checked by a second reviewer. We report covariate-adjusted associations when available.

### Quality Assessment

We used the STROBE checklist (www.equator-network.org) to score study methodology and assess study quality. We assigned up to 22 points using this checklist. This score was applied after study inclusion and did not influence whether a study was included in the review.

Owing to the small number of studies per category and the large methodological heterogeneity, it was not possible to combine study data by meta-analysis.

## Results

**Figure 1:**
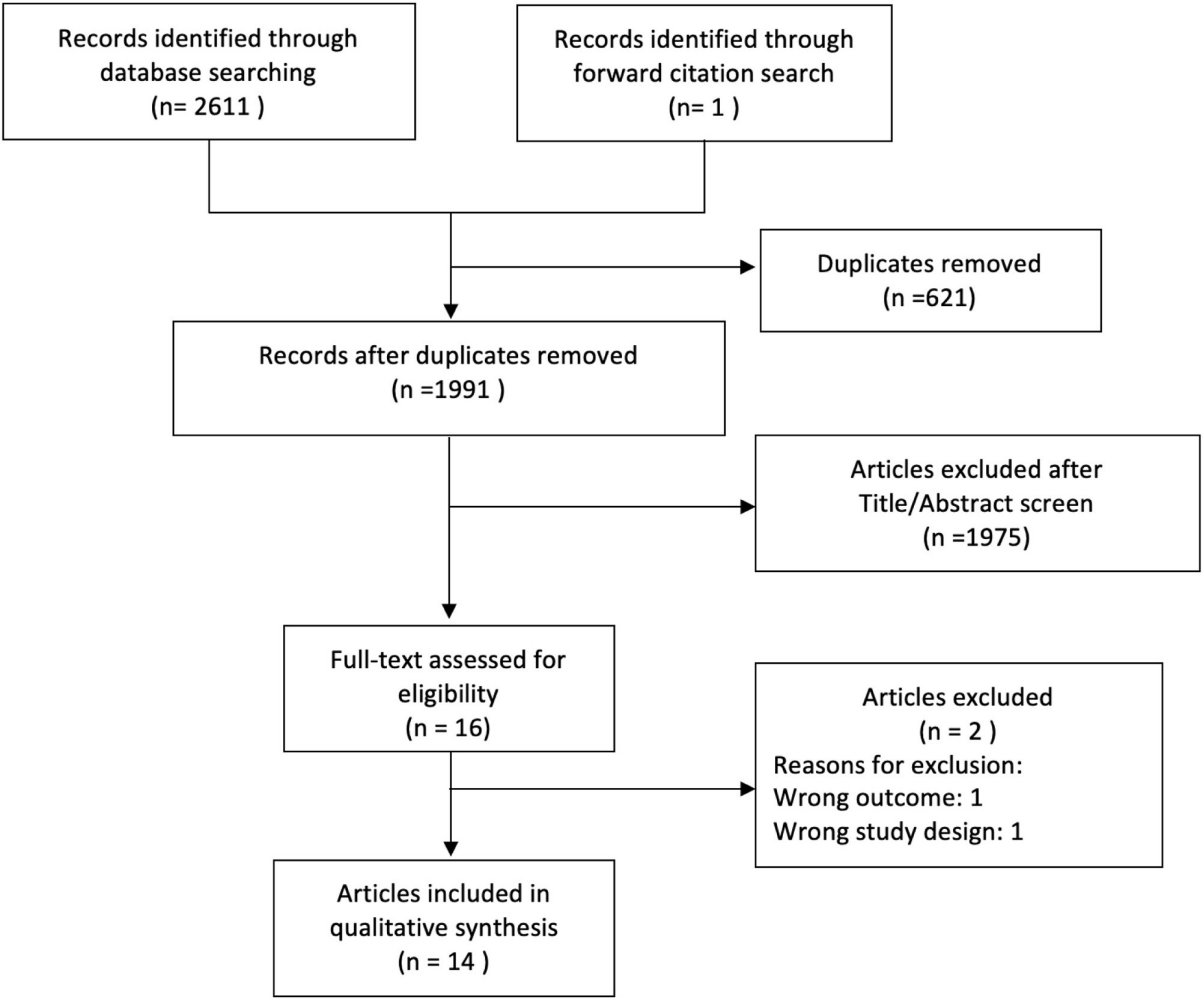
Search strategy flow chart.

The search returned 2,611 articles. One additional article was found by hand-search. Among them, 621 duplicate articles were removed. The remaining 1,991 articles were screened by title and abstract only. Of those, 16 were considered potentially relevant and were assessed by full-text review. Figure 1 details numbers of articles excluded and the reasons.

Fourteen articles met the inclusion criteria. The population samples came from the United States (five articles), the Netherlands (two articles), Germany (two articles), Italy (two articles), Poland (one article), Turkey (one article), and China (one article). Multiple articles from the same study population were included only if different retinal parameters or outcomes were measured in separate articles.

### Study Design and Patient Characteristics

Table 1 summarizes the study design and patient characteristics from the articles included. The 14 articles (14–27) included comprised 13 cross-sectional studies and 1 case–control study. Although two articles (18, 19) had overlapping samples, each study assessed different outcomes. Across the included studies (if two articles had an overlapping study population, the study with the larger sample was chosen), the total number of unique participants was 989. Participants in each study varied from 16 to 213 (median *n* = 55).

**Table 1:** Study design and patient characteristics.

**Article**	**Design of study**	**Number of subjects (eyes)**		**Eye selection**	**Mean age** **±** **SD (years)**		**Sex (Male/Female)**		**MMSE (Mean)**	
**AD studies**		**Patients**	**HC**		**Patients**	**HC**	**Patients**	**HC**	**Patients**	**HC**
Bulut et al. (14)	Cross-sectional study	AD 26 (26)	26 (26)	One eye from each subject was randomly selected	AD 74.2 ± 7.6	72.6 ± 6.3	AD 11/15	13/13	AD 17	27
Lahme et al. (15)	Cross-sectional study	AD 36 (36)	38 (38)	—	AD 68.0 ± 9.3	66.1 ± 10.1	AD 15/21	14/23	AD 22	—
Haan et al. (16)	Cross-sectional study	AD 48 (NA)	38	Values from both eyes were averaged unless only one eye was suitable	AD 65.4 ± 8.1	60.6 ± 5.0	AD 25/23	24/14	AD 23	29
Querques et al. (17)	Cross-sectional study	AD 12 (12) MCI 12 (12)	32 (32)	One eye for each subject was randomly selected	AD 72.9 ± 7.2 MCI 76.3 ± 6.9	71.6 ± 5.9	AD 4/8 MCI 5/7	17/15	AD 21 MCI 25	—
Yoon et al. (18)*	Cross-sectional study	AD 39 (70) MCI 41 (79)	133 (254)	Eye images with poor scan quality and motion artifact were excluded	AD 72.8 ± 7.7 MCI 71.1 ± 7.6	69.2 ± 7.8	AD 13/26 MCI 17/20	36/97	AD 20 MCI 23	29
Yoon et al. (19)*	Cross-sectional study	AD 9 (17) MCI 7 (13)	—	Values from both eyes were averaged unless only one eye was suitable	AD 75.2 ± 7.5 MCI 70.7 ± 9.1	—	AD 4/5 MCI 4/3	—	AD 22 MCI 26	—
Zabel et al. (20)	Cross-sectional study	AD 27 (27)	27 (27)	One eye of each patient was included	AD 74.1 ± 5.9	74.3 ± 7.7	AD 6/21	8/19	AD 21	28
Jiang et al. (21)	Cross-sectional study	AD 12 (NA) MCI 19 (NA)	21	Values from both eyes were averaged	AD 73.3 ± 9.6 MCI 69.6 ± 9.8	67.6 ± 8.3	AD 7/5 MCI 7/12	7/14	AD 20 MCI 26	30
Wu et al. (22)	Cross-sectional study	AD 18 (28) MCI 21 (32)	21 (33)	Eye images with poor scan quality and motion artifact were excluded	AD 69.9 ± 6.4 MCI 67.8 ± 6.0	68.7 ± 5.9	— MCI 12/9	11/10	AD 20 MCI 25	27
Zhang et al. (23)	Cross-sectional study	aMCI/eAD 16 (16)	16 (16)	Chose right eyes. If the image quality failed, then chose left eyes.	aMCI/eAD 73.03 ± 8.24	73.6 ± 7.7	aMCI/eAD 3/13	3/13	MoCA 20	MoCA 27
**Presymptomatic AD studies**										
van de Kreeke, et al. (24)	Cross-sectional study	Aβ+ 13	Aβ- 111	Values from both eyes were averaged	ALL 68.6 ± 6.3		ALL 58/66		ALL 29	
O'Bryhim et al. (25)	Case–control study	ALL 30 (58)		Eye images with poor scan quality and motion artifact were excluded	ALL 74.5 ± 5.6		ALL 14/16		(normal)	
**CADASIL study**		**CADASIL**	**HC**		**CADASIL**	**HC**	**CADASIL**	**HC**	**CADASIL**	**HC**
Nelis et al. (26)	Cross-sectional study	11 (21)	21 (21)	Values from both eyes were used.	53.5 ± 10.7	53.8 ± 11.5	3/8	—	—	—
**Fabry disease study**		**Fabry**	**HC**		**Fabry**	**HC**	**Fabry**	**HC**	**Fabry**	**HC**
Cennamo et al. (27)	Cross-sectional study	54 (54)	70 (70)	One eye for each subject was randomly selected	44.1 ± 15.6	42.3 ± 15.6	20/34	34/36	—	—

*AD, Alzheimer disease; MCI, mild cognitive impairment; HC, healthy control; eAD, early Alzheimer-type dementia; aMCI, amnestic type of MCI; MMSE, Mini-Mental State Examination; MoCA, Montreal Cognitive Assessment; CADASIL, cerebral autosomal dominant arteriopathy with subcortical infarcts and leukoencephalopathy*.

* *The two studies were from the same study population*.

**Figure 2:**
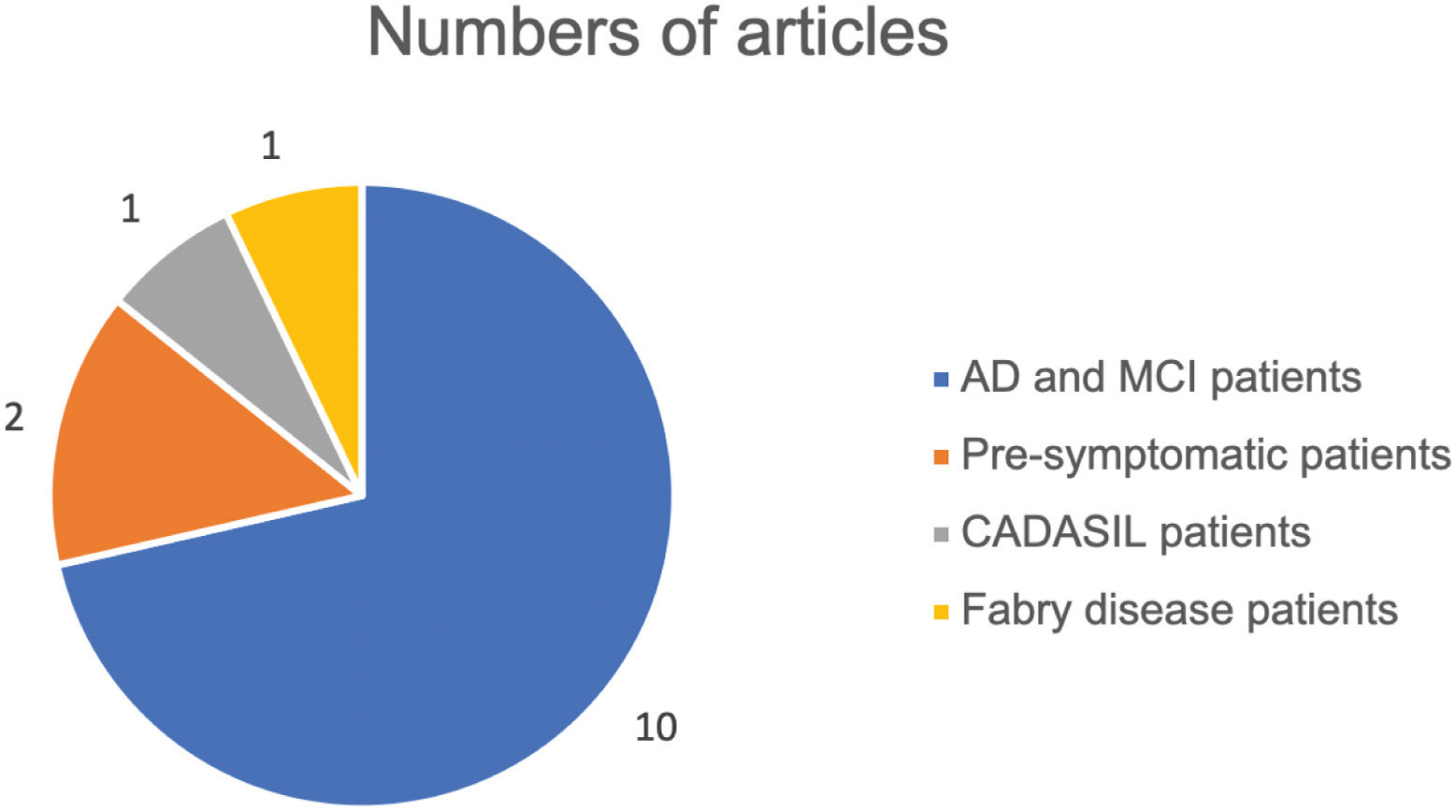
Study populations in reviewed studies. AD, Alzheimer disease; MCI, mild cognitive impairment; CADASIL, cerebral autosomal dominant arteriopathy with subcortical infarcts and leukoencephalopathy.

Ten articles (14–23) included patients with AD and mild cognitive impairment (MCI) (AD, *n* = 221; MCI, *n* = 106; controls, *n* = 352). Two studies (24, 25) investigated presymptomatic AD participants who had normal cognitive function with positive biomarker of amyloid-β (Aβ) (total *n* = 154). No relevant studies were identified for other types of dementia. Two studies investigated monogenic SVD patients: one (26) studied cerebral autosomal dominant arteriopathy with subcortical infarcts and leukoencephalopathy (CADASIL) patients (CADASIL, *n* = 11; controls, *n* = 21), and the other (27) studied Fabry disease (Fabry disease, *n* = 54; controls, *n* = 70). We did not find any study on sporadic SVD or ischemic stroke that met the inclusion criteria (Figure 2).

### Quality Assessment

The median study quality score as per STROBE checklist (www.equator-network.org) was 16/22 (Supplementary Table 1). Scores were mainly affected by inadequate description of study design in title or abstract, not explaining rationale for study size, inadequate description of efforts made to control potential sources of bias, and inadequate discussion of generalizability of results.

### OCTA Devices, Parameters, and Calculation Method

Table 2 summarizes the methods used by each study to assess retinal parameters. Eight articles (14, 15, 20, 22, 23, 25–27) used AngioVue™ system of Optovue, and the other six articles (16–19, 21, 24) used AngioPlex™ of Carl Zeiss. Different manufacturers use different hardware, software, and algorithms for vessel segmentation, which complicates interstudy comparisons, and indeed, OCTA parameters with the same name may be defined differently across studies. Furthermore, different studies used different image quality assessment methods or cutoffs (Supplementary Table 2). Therefore, the results from those studies are not all interchangeable (28). See Table 3 for detailed explanation of these retinal parameters.

**Table 2:** OCTA devices, parameters, and calculation method.

**Article**	**OCTA device**	**Macular scan size (mm)**	**ONH scan size (mm)**	**OCTA parameters**	**Additional adjustments**
Bulut et al. (14)	Optovue	6	—	VD (%): Whole/fovea/parafovea superficial VD FAZ (mm^2^) Flow index rates: outer retina/choroidal flow rate	None (age and sex matched)
Lahme et al. (15)	Optovue	3	4.5	VD (%): Whole/fovea/parafovea superficial VDWhole/fovea/parafovea deep VD Whole radial peripapillary capillaries/radial peripapillary capillaries VD	None (age matched)
Haan et al. (16)	Zeiss	6	—	VD (/mm): inner ring (Ø 1–3 mm around fovea) VD Outer ring (Ø 3–6 mm around fovea) VDFAZ (mm^2^)	Age, spherical equivalent, and quality factor
Querques et al. (17)	Zeiss	3 6	—	PD (%), 3 and 6 mm Superficial/deep vascular plexus -PD Choriocapillaris/choroida -PD	None (age and sex matched)
Yoon et al. (18)	Zeiss	3 6	—	Superficial vascular plexus of VD (/mm) and PD (%) in: 3-mm circle, 3-mm ring (Ø 1–3 mm around fovea), and 6-mm circle FAZ (mm^2^)	Age and sex
Yoon et al. (19)	Zeiss	3 6	—	VD (/mm) and PD (%):6 * 6 mm: 6-mm circle, 3-mm ring (Ø 1–3 mm around fovea) and 6-mm ring (Ø 3–6 mm around fovea) 3 *3 mm: 3-mm circle and 3-mm ring (Ø 1–3 mm around fovea) FAZ (mm^2^)	None
Zabel et al. (20)	Optovue	6	4.5	VD (%): Whole superficial/deep-VD,Whole radial peripapillary capillaries VD Radial peripapillary capillaries VD FAZ (mm^2^)	None
Jiang et al. (21)	Zeiss	3	—	VD (Dbox): Area: ring (Ø 0.6–2.5 mm around fovea), Further separate (a), into four quadrantal sectors(b), into six concentric ringsSegmentation: total, superficial and deep layers	None
Wu et al. (22)	Optovue	6	—	VD (%):1.5-mm ring (Ø 0.3–1.5 mm around fovea) 3-mm ring (Ø 1.5–3 mm around fovea) Each ring was separated into four quadrantal sectorsFAZ (mm^2^).	None (age and sex matched)
Zhang et al. (23)	Optovue	3	4.5	VD (%) and VLD (%): Parafoveal superficial capillary plexus VD/VLD, Peripapillary radial peripapillary capillary VD/VLD Peripapillary superficial vascular plexus VD/VLD Adjusted flow index: Parafoveal superficial capillary plexus	None (age, sex and race matched)
van de Kreeke, et al. (24)	Zeiss	6	—	VD (/mm): inner ring (Ø 1–3 mm around fovea) VD outer ring (Ø 3–6 mm around fovea) VD ONH ring (Ø 3–6 mm around the ONH) VD FAZ (mm^2^)	Age
O'Bryhim et al. (25)	Optovue	6	—	FAZ (mm^2^)	Age, gender, scan quality, and diagnosis of diabetes
Nelis et al. (26)	Optovue	3	4.5	FAZ (mm^2^) (separated into superficial and deep layers)VD (%): Parafoveal superficial/deep VD and OND VDChoriocapillaris [CC] parameters: Choriocapillaris (CC) decorrelation index (%) Choriocapillaris flow area (%) Analysis of signal voids	None (age matched)
Cennamo et al. (27)	Optovue	6	—	VD (%): Superficial AND Deep capillary plexus: Whole image/parafovea/fovea VD	Age and sex

*AD, Alzheimer disease; MCI, mild cognitive impairment; HC, healthy control; eAD, early Alzheimer-type dementia; aMCI, amnestic type of MCI; CADASIL, Cerebral autosomal dominant arteriopathy with subcortical infarcts and leukoencephalopathy, OCTA, optical coherence tomography angiography; ONH, optic nerve head; VD, vessel density; VLD, vessel length density; PD, perfusion density; FAZ, foveal avascular zone; ANOVA, analysis of variance*.

**Table 3:** OCTA retinal parameters assessed by reviewed studies.

**OCTA retinal parameters**	**Descriptions**	**Study**
Foveal avascular zone (FAZ) (mm^2^)	The central avascular region in human foveola is known as the foveal avascular zone (FAZ)	(14, 16, 18–20, 22, 24–26)
Perfusion density (PD) (%)	In most Carl Zeiss devices, PD (%) was defined as the total area of perfused retinal microvasculature per unit area in a region of measurement	(17–19)
Vessel density or vascular density (VD)	In most Optovue devices, VD (%) has the similar definition of PD in Carl Zeiss device	(14, 15, 20, 22, 23, 26, 27)
	In most Carl Zeiss devices, VD (/mm) was defined as the total length of perfused retinal microvasculature per unit area in the region of measurement	(16, 18, 19, 24)
	VD (Dvox) is analyzed using fractal analysis with box counting (Dbox)	(21)
Vessel length density (VLD)	VLD (%) (using Optovue device) represents the ratio of the total length occupied by all the blood vessels in a given area to the total area. All vessels were then skeletonized into 1-pixel-wide vessels	(23)
Adjusted flow index or flow index rate or decorrelation signal index	Adjusted flow index, a surrogate measure of blood flow velocity in OCTA, is defined as the average decorrelation value of all pixels above the noise threshold (only “vessels”) in the *en face* angiogram. Adjusted flow index and flow index rate have no unit, while the decorrelation signal index is “%”	(14, 23, 26)
Choriocapillaris flow area (%)	The choriocapillaris vascular flow area was defined as the percentage of the portion of white pixels against the whole scan area	(26)
Analysis of signal voids in choriocapillaris area (μm^2^)	The proportion of absent flow signal accounted for by signal voids >10,000 μm^2^ (FV10000) and signal voids >40,000 μm^2^ (FV40000) were evaluated	(26)

### Main Analysis

#### OCTA Parameters in Patients vs. Controls

**Figure 3:**
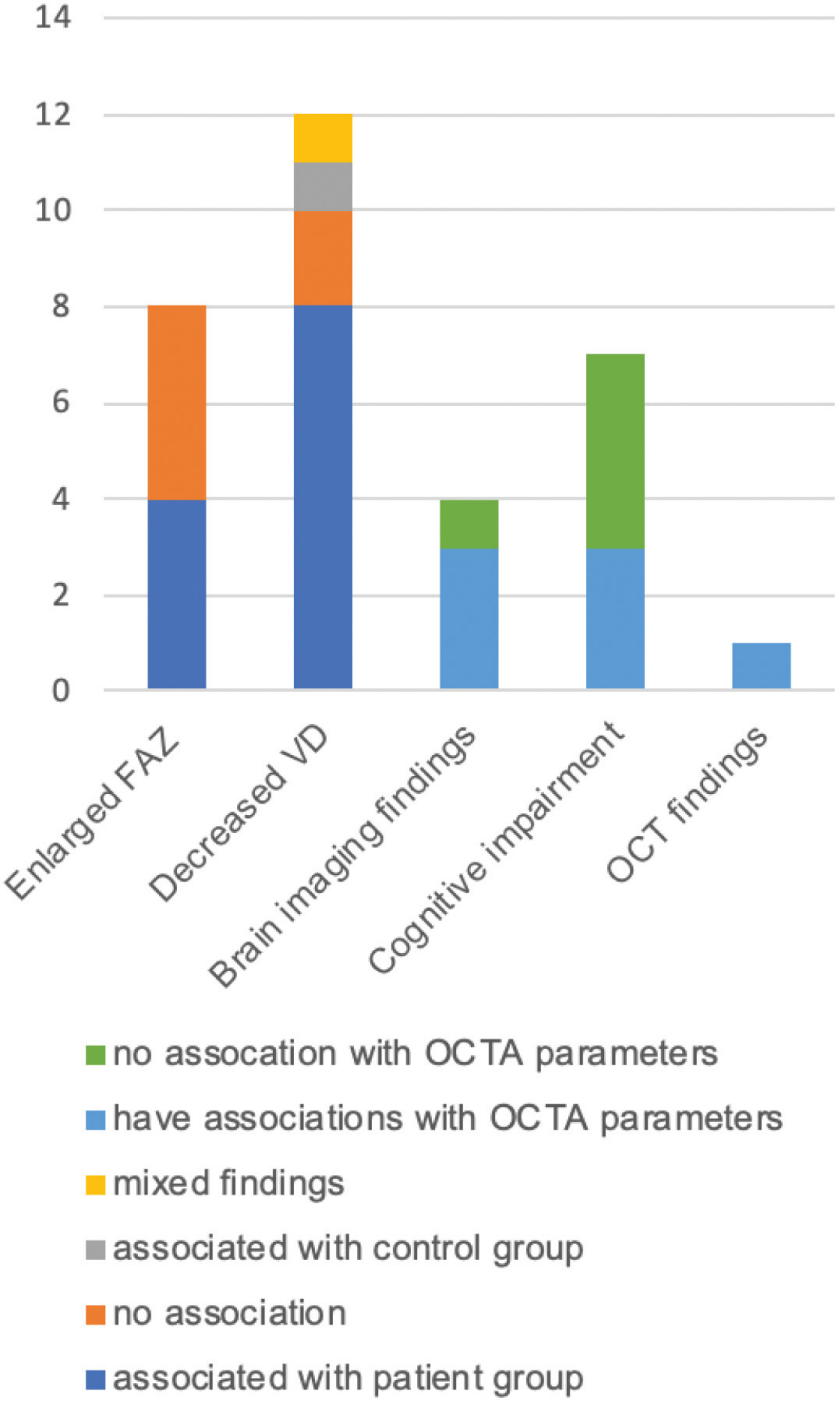
Finding summaries of reviewed studies. OCTA, optical coherence tomography angiography; OCT, optical coherence tomography; FAZ, foveal avascular zone; VD, vessel density.

Figure 3 and Table 4 summarize the main results from the 14 included studies. Eight studies (14, 16, 18, 20, 22, 24–26) compared the foveal avascular zone (FAZ) size between patients and controls. Four studies [*n* = 196, median *n* = 53, three AD and MCI studies (14, 20, 22); one presymptomatic AD study (25)] found that patients had enlarged FAZ compared with controls. Among these studies, two (14, 22) were age- and sex-matched; another (25) adjusted for age, sex, scan quality, and diabetes; and the other (20) did not adjust for any covariates. Four other studies [*n* = 455, median n = 105, two AD and MCI studies (16, 18); one presymptomatic AD study (24); one CADASIL study (26)] showed no difference between patient and control groups. Among these studies, one study matched for age and sex (18). Two studies (16, 24) adjusted for age, one (16) of which also adjusted for spherical equivalent, and quality factor. The other study (26) did not adjust for any covariates.

**Table 4:** Results of included studies.

**Article**	**OCTA parameters in subjects vs. controls**	**OCTA and brain imaging findings**	**OCTA and cognitive function**	**OCTA and fundus or OCT images**
Bulut et al. (14) (*n* = 52)	The group of patients with AD had lower VD and enlarged FAZ area compared with the control group	Not assessed	Increased MMSE score associated with all increased VD parameters and decreased FAZ area	Not assessed
Lahme et al. (15) (*n* = 74)	The group of patients with AD had lower VD compared with control group	Increased VD associated with decreased Fazekas score	No association between MMSE score and VD	Not assessed
Haan et al. (16) (*n* = 86)	No difference between AD and control groups	Increased VD associated with decreased Fazekas score	No association between MMSE score and OCTA parameters	Not assessed
Querques et al. (17) (*n* = 56)	No differences were found among groups	Not assessed	Not assessed	Not assessed
Yoon et al. (18) (*n* = 213)	The AD group showed reduced VD and PD compared with MCI group and control group No difference between MCI and control groups	Not assessed	Increased MMSE score associated with increased VD and PD parameters No association between MMSE score and FAZ	Not assessed
Yoon et al. (19) (*n* = 16)	Not assessed	Decreased VD and PD with increased volume of inferolateral ventricle	Not assessed	Not assessed
Zabel et al. (20) (*n* = 54)	AD group showed reduced VD and increased FAZ compared with control group	Not assessed	No association between MMSE score and OCTA parameters	Not assessed
Jiang et al. (21) (*n* = 52)	AD group showed reduced VD compared with control group	Not assessed	No association between MMSE score and VD	Increased VD associated with increased GC-IPL thickness in AD patients
Wu et al. (22) (*n* = 60)	The patient groups had lower VD and enlarged FAZ area compared with the control group	Not assessed	Not assessed	Not assessed
Zhang et al. (23) (*n* = 32)	The aMCI/eAD group showed decreased VD and adjusted flow index compared with control group No differences in vessel length density and FAZ between two groups	Not assessed	Increased MoCA score associated with increased VD and vessel length density	Not assessed
van de Kreeke et al. (24) (*n* = 124)	Aβ+ participants had higher VD than Aβ- participants No differences in FAZ between two groups	Not assessed	Not assessed	Not assessed
O'Bryhim et al. (25) (*n* = 30)	The FAZ was increased in the biomarker-positive group compared with controls	Not assessed	Not assessed	Not assessed
Nelis et al. (26) (*n* = 32)	CADASIL group showed decreased VD compared with control group No differences in FAZ and choriocapillaris parameters between groups	No association between OCTA parameters and Fazekas score and number of small infarcts	Not assessed	Not assessed
Cennamo et al. (27) (*n* = 124)	Fabry disease group showed decreased VD in superficial capillary plexus compared with control group Fabry disease group showed increased VD in deep capillary plexus compared with control group	Not assessed	Not assessed	Not assessed

*AD, Alzheimer disease; MCI, mild cognitive impairment; HC, healthy control; MMSE, Mini-mental State Examination; MoCA, Montreal Cognitive Assessment; OCTA, Optical coherence tomography angiography; OCT, Optical coherence tomography; FAZ, Foveal avascular zone; VD, vessel density; PD, perfusion density; VLD, Vessel length density; CADASIL, Cerebral autosomal dominant arteriopathy with subcortical infarcts and leukoencephalopathy; GC-IPL, ganglion cell–inner plexiform layer*.

Twelve studies (14–18, 20–24, 26, 27) compared the vascular density parameters (vessel density, vessel length density, perfusion density, etc.) between patients and controls. From these, eight studies [*n* = 569, median *n* = 53, seven AD and MCI studies (14, 15, 18, 20–23); one CADASIL study (26)] suggested decreased vascular density parameters in patients compared with controls. Among them, two studies (20, 21) did not adjust for any covariates, one study (18) adjusted the results for age and sex, and in four studies (14, 15, 23, 26), sample groups were matched for age, three of which (14, 22, 23) also matched for sex and one of them (23) also matched for race. However, two studies [*n* = 56 and *n* = 86, involving AD and MCI patients (16, 17)] showed no difference between patient and control groups. Interestingly, one study in presymptomatic AD (*n* = 124) (24) found Aβ+ participants had higher vessel density than Aβ- participants after adjusting for age. Another study (*n* = 124) (27) found Fabry disease patients had decreased vascular density in superficial capillary plexus and increased in deep capillary plexus compared with controls after adjusting for age and sex.

#### OCTA Parameters in Relation to Brain Imaging Findings

Only four studies [*n* = 208, median *n* = 53, three in AD and MCI (15, 16, 19) and one in CADASIL (26)] investigated the relationship between OCTA findings and brain imaging findings. Two of them (*n* = 74 and *n* = 86) (15, 16) involving AD and MCI participants found increased vessel density (VD) was associated with having fewer white matter hyperintensities (WMHs), as per Fazekas scores. Between these two studies, one (15) matched for age, and the other (16) adjusted for age and sex. One study (*n* = 16) (19) involving AD and MCI patients suggested a potential association between decreased VD and decreased perfusion density with increased volume of the inferior lateral ventricle (temporal horn), but it did not control for covariates, and patients and controls were not matched by age and/or gender. The study (*n* = 32) (26) in CADASIL patients found no association between OCTA parameters and the number of small infarcts or WMHs, the latter estimated using Fazekas scores.

#### OCTA Findings in Relation to Cognitive Function

Seven (14–16, 18, 20, 21, 23) studies investigated the relationship between OCTA parameters and cognitive function in AD and MCI participants. Three studies (*n* = 297) (14, 18, 23) suggested better cognitive function was associated with increased VD. One study (*n* = 52) (14) found enlarged FAZ area was associated with decreased Mini-Mental State Examination (MMSE) scores. Of these three studies, two (14, 23) were age- and sex-matched, one of which (23) was also race-matched. One study (18) did not adjust or match for covariates. However, four other studies (*n* = 266, median *n* = 64) (15, 16, 20, 21) found no association between OCTA parameters and cognitive functions. Among them, one study (16) found no association, both adjusted and unadjusted for age and sex, whereas the other three studies (15, 20, 21) did not match or adjust for covariates.

#### OCTA Parameters and Other OCT/Fundus Imaging Parameters

Only one study (*n* = 52) (21) explored the relationship between OCTA features and OCT findings in patients with AD and found that increased VD was associated with increased ganglion cell–inner plexiform layer (GC-IPL) thickness. However, this study did not adjust or match for any covariates. None of the studies included investigated the relationship between OCTA parameters and traditional fundus imaging.

## Discussion

This systematic review found 14 studies investigating OCTA in patients with cerebral SVD or dementia, published between 2017 and 2020. These studies mainly focused on AD and MCI, pre-symptomatic AD, and monogenic SVD including CADASIL and Fabry disease. No other dementias, ischemic stroke, or sporadic SVD studies met the inclusion criteria. There were no longitudinal studies. Collectively, these studies do not provide a clear conclusion of whether the retinal microvasculature is impaired in cerebral SVD, ischemic stroke, and dementia patients or not.

FAZ is a capillary-free area in the foveal zone susceptible to ischemia (29). Enlargement of FAZ is a sign of ischemia (14). The analysis of the FAZ measurements yielded inconclusive results. Four studies (*n* = 196) (14, 20, 22, 25) found increased FAZ in patients compared with controls, whereas four other studies (*n* = 455) (16, 18, 24, 26) showed no difference between groups. It is possible that the relatively small sample size of some studies precludes detection of group differences. Another possibility is that some studies included patients in the earlier stage of the disease process, which might explain why no FAZ difference was found between groups (24). However, as ophthalmological confounders, quality factors, and other possible risk factor confounders including age were not always taken into account, studies reporting group differences may represent an overestimation of true disease effects (16). Furthermore, significant variation in FAZ area in normal eyes has been reported that may be associated with gender, central retinal thickness, etc. (30). Therefore, studies with larger sample size and good matching of potential confounders are needed to further verify these findings. Also, the lack of longitudinal studies means that it is difficult to know if the early OCTA changes could predate worsening neurological state, and longitudinal studies are urgently needed.

The decreased vascular density (including parameters such as VD, perfusion density, vessel length density, etc.) is another sign of microvasculature impairment. Eight studies (*n* = 569, seven with AD and MCI and one with CADASIL) (14, 15, 18, 20–23, 26) suggested decreased vascular density parameters in patients compared with controls. However, two studies (*n* = 56–86) (16, 17) showed no difference between groups. Different sample size, disease stage, and confounder adjustment might explain the disparity in results. Interestingly, van de Kreeke et al. (*n* = 124) (24) found Aβ+ participants had higher VD than Aβ- participants in pre-symptomatic AD. Here, early amyloid accumulation in the retina might induce an inflammatory reaction with hypoxia, leading to increased retinal blood flow (24). Larger studies including those following patients longitudinally will help explore retinal vessel changes. Moreover, Cennamo et al. (*n* = 124) (27) found Fabry disease patients had decreased VD in superficial capillary plexus and increased VD in deep capillary plexus compared with the control group. The authors considered the increased VD in deep capillary plexus as a compensatory mechanism to support the reduced VD in superficial capillary plexus (27).

We further explored the relationships between OCTA and brain imaging findings. Currently, cerebral small vessels are hard to visualize *in vivo*. Other imaging features are provided as SVD biomarkers such as WMHs, microbleeds, enlarged perivascular spaces, and lacunes (1). Because the retinal vasculature is a proxy of cerebral vessels (4), establishing the relationship between retinal microvasculature and cerebral imaging findings could aid in our understanding of disease mechanisms. This systematic review found only four studies (15, 16, 19, 26) that investigated the relationship between OCTA parameters and brain imaging findings of SVD. Two studies (*n* = 74–86) (15, 16) found decreased VD was associated with more WMHs, whereas another study (*n* = 32) (26) found no association between OCTA parameters and WMHs or the number of small infarcts, which might be due to small sample size. MRI changes, such as atrophy of the brain and consequential ventricular enlargement, can be seen in AD or SVD patients (31). A pilot study (*n* = 16) (19) suggested the potential association between decreased VD and perfusion density with increased volume of inferior lateral ventricle (temporal horn). These small sample studies show a promising role of OCTA to investigate SVD *in vivo*. A prospective cross-sectional study (32) published recently when our work was under review investigating the amyloid-positive AD-related cognitive impairment (*n* = 28), subcortical vascular cognitive impairment (*n* = 18) patients, and amyloid-negative cognitively normal subjects (*n* = 14) found that VD was negatively correlated with SVD scores and suggested OCTA as a potential imaging tool to screen for the degree of SVD. Further studies in larger samples comparing a range of SVD brain imaging biomarkers with OCTA parameters are needed to confirm relationships and explore the underlying pathophysiological mechanisms in SVD.

Previous studies reported that the changes in retinal vessel caliber on fundus imaging were associated with cognitive decline (5). This systematic review found seven studies investigated the relationship between OCTA findings and cognitive function. Among them, three studies (*n* = 297) (14, 18, 23) suggested better cognitive function was associated with higher VD. One study (*n* = 52) (14) found enlarged FAZ area was associated with decreased MMSE score. However, four other studies (*n* = 266) (15, 16, 20, 21) found no association between OCTA parameters and cognitive function. A cross-sectional study (33) in 27 amnestic mild cognitive impaired patients and 29 controls published after completing this review did not find any correlation between MMSE scores and OCTA parameters. Contradictory results, relatively small samples with limited or no adjustment for covariates, and no long-term follow-up of the studies that suggest OCTA parameters as potential predictors of cognitive decline point to the necessity of larger, longitudinal studies in this area of research.

We found only one study (*n* = 52) (21) investigating the relationship between OCTA features and OCT findings that suggested increased VD was associated with increased GC-IPL thickness in AD patients. However, this study did not adjust for covariates. The optic nerve and retina develop as a direct extension of the diencephalon during embryonic development (4). OCT is a non-invasive method and offers high-resolution images of the retinal structure, including the neuronal layers (6). Exploring the relationship between OCT and OCTA findings especially in longitudinal studies will not only provide evidence of the temporal changes between retinal nerve layers and retinal microvasculature, but also offer potential predictors in disease progression.

We did not find any study investigating the relationship between OCTA parameters and fundus imaging. Retinal arterioles and venules typically measure 15 to 150 μm in size, whereas the capillaries measure 5 to 15 μm (34). There is an extensive literature studying the retinal vessel changes in SVD, ischemic stroke, and dementia using fundus imaging (5, 35). However, recent fundus imaging can only measure relatively large retinal vessels, whereas OCTA provides high-resolution, depth-resolved information that has been available only since 2016 (8). The relationships between changes in the relatively larger vessels and smaller vessels seen only on OCTA are not fully understood. Further studies investigating the relationship between these two measurements are required. It should also be noted that longitudinal studies assessing fundus images and future neurological outcomes are also limited pointing to the need for more longitudinal studies of OCTA and fundus images in SVD.

The methodological differences limit between-study comparisons and interpretation. The variations in the different device types, software and hardware, macular scan size, segmentation methods, and retinal parameters all add to heterogeneity. Other potentially confounding factors such as age, race, and baseline status including diagnosis of diabetes are inevitable. The asymmetry between eyes of the same individual should be considered because it might cause potential misleading results when we choose one eye as a proxy for both eyes (36). On the contrary, studies utilizing both eyes of participants should address the statistical challenges cautiously because the pair of eyes are non-independent (36). OCTA scan quality has impacts on derived parameters, and researchers should perform strict quality control to ensure good data quality, and thus results are reliable and comparable. Patients with more severe disease status may have fixation problems, which may cause poor scan quality (14). Lastly, studies reviewed here were cross-sectional, and no conclusions can be drawn on the value of OCTA measurements for evaluation of disease progression.

## Conclusions

The findings of this review show promise that changes in retinal microvasculature identified using OCTA are associated with monogenic SVD and different stages of AD. Larger studies with risk factors adjustment and more consistent OCTA methods are needed to fully exploit this technology.

## Data Availability Statement

All datasets generated for this study are included in the article/Supplementary Material.

## Author Contributions

J-FZ, SW, and MV-H searched the literature, drafted the manuscript, collected the data, and performed statistical analyses. J-FZ, SW, MV-H, FD, BD, Y-CW, and JW revised the manuscript. J-FZ, SW, MV-H, Y-CW, and JW contributed to conception, design, and data interpretation of the study. SW, MV-H, FD, BD, Y-CW, and JW contributed to manuscript revision for critical intellectual content and supervision of the study. All authors read and approved the manuscript.

## Conflict of Interest

The authors declare that the research was conducted in the absence of any commercial or financial relationships that could be construed as a potential conflict of interest.

## Glossary

OCTA, optical coherence tomography angiography; SVD, small vessel disease; AD, Alzheimer disease; MCI, mild cognitive impairment; CADASIL, cerebral autosomal dominant arteriopathy with subcortical infarcts and leukoencephalopathy; VD, vessel density, OCT, optical coherence tomography; FAZ, foveal avascular zone; WMHs, white matter hyperintensities; MMSE, Mini-Mental State Examination.
