# Supplementary material for: The Application of Optical Coherence Tomography Angiography in Cerebral Small Vessel Disease, Ischemic Stroke, and Dementia: A Systematic Review
Source: Front Neurol. 2020 Sep 10;11:1009. doi: 10.3389/fneur.2020.01009 (PMC7511809; doi:10.3389/fneur.2020.01009)
Supplement: Supplementary file 1 [file Table_1.DOCX]

**Supplementary materials**

OCTA and SVD systematic review search strategy

Search strategy

1. brain ischemia/ or brain infarction/ or brain stem infarctions/ or cerebral infarction/ or hypoxia-ischemia, brain/ or stroke/

2. (isch?emi* adj6 (stroke* or apoplexy* or cerebral vasc* or cerebrovasc* or cva or attack*)).tw.

3. ((brain or cerebr* or cerebell* or vertebrobasil* or hemisphere* or intracran* or intracerebral or infratentorial or supratentorial or middle cerebr* or mca* or anterior circulation) adj5 (isch?emi* or infarct* or thrombo* or emboli* or occlus* or hypoxi*)).tw.

4. (lacun* or small vessel* or small infarct* or microinfarct* or subcortical lesion* or subcortical infarct* or microvascular* or microcirculation*).tw.

5. 1, 2, 3, OR 4.

6. (Dement* or memory or Alzheimer* or AD or cognitive or cognition or MCI or mild cognitive impairment or mild neurocognitive disorder).mp.

7. (vascular dementia or VD or VCI or VaD).mp.

8. (Lewy bod* or LBD or frontotemporal or FTD).mp.

9, 6, 7, OR 8.

10. (MR or magnetic resonance or structural imag* or CT or computed tomograph*).mp.

11. (small vessel disease* or small-vessel or SVD or microvascular change).mp.

12. (((white matter or white-matter) AND (hyperintensit* or lesion* or disease* or change* or abnormalit*)) or WMH or leukoaraiosis).mp.

13. (micro-bleed* or microbleed* or microh?emorr*).mp.

14. (perivascular space* or PVS or Virchow-Robin space* or VRS).mp.

15. (volume loss or atrophy).mp.

16. 10, 11, 12, 13, 14, OR 15.

17. 5, 9, OR16.

18. (OCTA or optical coherence tomography angiography or OCT-A or OCT-angiography or OCT angiography or optical coherence tomographic angiography).mp.

19. 17 AND 18.

20. limit 19 to English language

Table S1 Assessment of STROBE criteria

| No |  | Bulut et al. (2018) | Lahme et al. (2018) | Haan et al. (2019) | Querques et al. (2019) | Yoon et al. (2019a) | Yoon et al.(2019b) | Zabel et al. (2019) | Jiang et al. (2017) | Wu et al. (2020) | Zhang et al. (2019) | van de Kreeke, et al (2019) | O’Bryhim et al. (2018) | Nelis et al. (2018) | Cennamo et al (2019) |
| --- | --- | --- | --- | --- | --- | --- | --- | --- | --- | --- | --- | --- | --- | --- | --- |
| 1 | Did the study indicate its design with a commonly used term in the title or the abstract, and provide in the abstract an informative and balanced summary of what was done and what was found? | **0** | **0** | **0** | **0** | **1** | **0** | **1** | **0** | **0** | **0** | **0** | **1** | **0** | **0** |
| 2 | Did the study explain the scientific background and rationale for the investigation being reported? | **1** | **1** | **1** | **1** | **1** | **1** | **1** | **1** | **1** | **1** | **1** | **1** | **1** | **1** |
| 3 | Did the study state specific objectives, including any prespecified hypotheses? | **1** | **1** | **1** | **1** | **1** | **1** | **1** | **1** | **1** | **1** | **1** | **1** | **1** | **1** |
| 4 | Did the study present key elements of study design early in the paper? | **0** | **0** | **0** | **1** | **1** | **0** | **1** | **0** | **1** | **0** | **0** | **1** | **0** | **0** |
| 5 | Did the study describe the setting, locations, and relevant dates, including periods of recruitment, exposure, follow-up, and data collection? | **0** | **0** | **0** | **1** | **1** | **1** | **1** | **0** | **1** | **0** | **1** | **1** | **1** | **1** |
| 6 | Did the study give the eligibility criteria, and the sources and methods of selection of participants? | **1** | **1** | **1** | **1** | **1** | **1** | **1** | **0** | **1** | **1** | **1** | **1** | **1** | **1** |
| 7 | Did the study clearly define all outcomes, exposures, predictors, potential confounders, and effect modifiers? (Give diagnostic criteria, if applicable) | **0** | **0** | **0** | **0** | **1** | **1** | **1** | **1** | **1** | **1** | **0** | **1** | **0** | **1** |
| 8 | For each variable of interest, did the study give sources of data and details of methods of assessment (measurement)? | **1** | **1** | **1** | **1** | **1** | **1** | **1** | **1** | **1** | **1** | **1** | **1** | **1** | **1** |
| 9 | Did the study describe any efforts to address potential sources of bias? | **0** | **0** | **1** | **0** | **1** | **1** | **1** | **0** | **0** | **0** | **1** | **1** | **0** | **1** |
| 10 | Did the study explain how the study size was arrived at? | **0** | **0** | **0** | **0** | **0** | **0** | **0** | **0** | **0** | **0** | **0** | **0** | **0** | **0** |
| 11 | Did the study explain how quantitative variables were analyzed? | **1** | **1** | **1** | **1** | **1** | **1** | **1** | **1** | **1** | **1** | **1** | **1** | **1** | **1** |
| 12 | Did the study describe all statistical methods? | **1** | **1** | **1** | **1** | **1** | **1** | **1** | **1** | **1** | **1** | **1** | **1** | **1** | **1** |
| 13 | Did the study report the number of participants in each part of the study and reasons for exclusions | **1** | **1** | **1** | **1** | **1** | **1** | **1** | **1** | **1** | **1** | **1** | **1** | **1** | **1** |
| 14 | Did the study report the descriptive data for study participants? | **1** | **1** | **1** | **1** | **1** | **1** | **1** | **1** | **1** | **1** | **1** | **1** | **1** | **1** |
| 15 | Did the study report the outcome data? | **1** | **1** | **1** | **1** | **1** | **1** | **1** | **1** | **1** | **1** | **1** | **1** | **1** | **1** |
| 16 | Did the study report the main results | **1** | **1** | **1** | **1** | **1** | **1** | **1** | **1** | **1** | **1** | **1** | **1** | **1** | **1** |
| 17 | Did the study report other analyses performed? —eg analyses of subgroups and interactions, and sensitivity analyses. | **1** | **1** | **1** | **1** | **1** | **1** | **1** | **1** | **1** | **1** | **1** | **1** | **1** | **1** |
| 18 | Did the study summarize key results with reference to study objectives? | **1** | **1** | **1** | **1** | **1** | **1** | **1** | **1** | **1** | **1** | **1** | **1** | **1** | **1** |
| 19 | Did the study discuss the limitations of the study taking into account sources of potential bias? | **0** | **0** | **0** | **0** | **0** | **0** | **1** | **0** | **0** | **0** | **0** | **0** | **0** | **0** |
| 20 | Did the study give a cautious overall interpretation of results considering objectives, limitations, multiplicity of analyses, results from similar studies, and other relevant evidence? | **1** | **1** | **1** | **1** | **1** | **1** | **1** | **1** | **1** | **1** | **1** | **1** | **1** | **1** |
| 21 | Did the study discuss the generalizability of results | **0** | **0** | **0** | **1** | **0** | **0** | **0** | **0** | **0** | **0** | **1** | **1** | **0** | **0** |
| 22 | Did the study give the source of funding? | **1** | **1** | **1** | **0** | **1** | **1** | **0** | **1** | **1** | **1** | **1** | **1** | **1** | **0** |
|  | Total score | **14** | **14** | **15** | **16** | **19** | **17** | **19** | **14** | **17** | **15** | **17** | **20** | **15** | **16** |

Table S2 OCTA image quality assessment mentioned in reviewed studies

| Article | Image quality assessment | Comments |
| --- | --- | --- |
| Yoon2019 a  Yoon2019 b | OCTA images that were of poor scan quality (less than 7/10 signal strength) because of low resolution or poor saturation and those that exhibited motion artifacts because of poor cooperation were excluded. | NA |
| Haan2019 | All scans had quality factors >7/10 and could be considered “good” quality. | Quality factors were included in adjustment. |
| Querques 2019 | Authors checked the quality of all images and only eyes with OCTA images of sufficient quality were included in the image processing. | NA |
| Bulut 2019 | For the scans to be included in data analysis, the requirement was to have a signal strength of a minimum of 60. | NA |
| Lahme 2018 | Only OCT-A images of good quality were included, and images with lines or gaps due to poor signal strength or motion artefacts were excluded from the study. | There was no significant difference between the signal strength index in the AD group and the control group. |
| Zabel 2019 | Only measurements of good technical quality with a signal quality (SQ) of 6 or more on a 10-degree scale, with which a commercial camera is equipped, qualified for further analysis. | NA |
| Jiang 2017 | NA | To ensure image quality, the cutoff of the signal strength of OCT was set to be 5, which is likely the minimal signal strength for OCT measurements of macular, optic nerve head, and retinal nerve fiber layer parameters. |
| Zhang2019 | All eyes included also had a signal quality measure (SQ) of ≥6, an absolute signal strength index (SSI) of ≥50 for the macula and SSI ≥45 for the disc, and no significant movement or shadow artifacts. The SQ is an integrated score of SSI as well as motion and shadow artifacts. | NA |
| Nelis 2018 | Images showing inadequate signal (signal strength index [SSI] < 50) or an OCT-A motion artifact score of three or four were excluded | NA |
| Cennamo2019 | Poor-quality images with a signal strength index (that reflects OCT image quality) of less than 40 or registered image sets with residual motion artefacts were excluded from the analysis. | NA |
| Van 2019 | Low-quality scans were excluded. | NA |
| O’Bryhim 2018 | NA | Four images were excluded owing to motion artifact or segmentation error; an additional 6 images were excluded owing to poor automated mapping that did not accurately represent the optic nerve disc or FAZ. |
| Wu 2020 | For acquiring clear images, the signal strength index was set at >40 for each eye. | NA |
